# The lncRNA Fer1L4 is an adverse prognostic parameter in clear-cell renal-cell carcinoma

**DOI:** 10.1007/s12094-020-02291-0

**Published:** 2020-01-21

**Authors:** A. Cox, Y. Tolkach, G. Kristiansen, M. Ritter, J. Ellinger

**Affiliations:** 1 Department of Urology, University Hospital Bonn, Bonn, Germany; 2 Institute of Pathology, University Hospital Bonn, Bonn, Germany

**Keywords:** Renal-cell carcinoma, Fer1L4, PCR, lncRNA, Survival

## Abstract

**Purpose:**

Long non-coding RNAs (lncRNA) are involved in oncogenesis and tumor progression in various tumor entities. At present, little is known about the role in tumor biology of the lncRNA Fer-1 like family member 4 (Fer1L4) in clear-cell renal-cell carcinoma (ccRCC). The aim of this study is to evaluate the expression of Fer1L4 in patients with ccRCC, its association with clinicopathological parameters, and value as prognostic biomarker.

**Material and methods:**

The expression of Fer1L4 was analyzed in the TCGA ccRCC cohort (*n* = 603; ccRCC *n* = 522, normal *n* = 81) and subsequently validated by quantitative real-time PCR in an independent cohort (*n* = 103, ccRCC *n* = 69, normal *n* = 34). Expression profiles were statistically correlated with clinicopathological and survival data.

**Results:**

Fer1L4 lncRNA is overexpressed in ccRCC compared to adjacent normal tissues. Increased expression significantly correlates with tumor aggressiveness: high expression levels of Fer1L4 RNA were found in higher grade, higher stage, and metastatic tumors. Furthermore, Fer1L4 overexpression is an independent prognostic factor for overall, cancer-specific, and progression-free survival of patients with ccRCC.

**Conclusion:**

Fer1L4 expression significantly correlates with aspects of tumor aggressiveness. Based on this impact on tumor progression and its influence as an independent prognostic factor, Fer1L4 appears to exert properties as an oncogene in ccRCC. As a prognostic tissue biomarker, further functional investigations are warranted to investigate Fer1L4 as a potential therapeutic target.

## Introduction

Long non-coding RNAs (lncRNAs) are defined as transcripts with a length > 200 nucleotides that are not transcribed into proteins [1]. The expression level of lncRNAs is about tenfold lower compared to the expression of mRNAs with markedly higher tissue specificity [2]. The aberrant expression level of lncRNAs is attributed to a high cell-to-cell variation in the expression of lncRNA genes in different cells [3, 4]. They are involved in transcriptional regulation of gene expression and thus convey their function in cell biology. Previous studies have shown that altered lncRNA expression has an effect on oncogenesis whereby they are emerging as potent regulators of tumor development [5–8]. Expression of different types of lncRNAs has also been reported in ccRCC [9, 10].

Recently, the lncRNA Fer-1 like family member 4 (Fer1L4) was shown to play a role in tumor biology. It was demonstrated that Fer1L4 influences cell proliferation as a tumor suppressor by acting as a competing endogenous RNA [11–13]. In various tumor entities such as gastric cancer, ovarian cancer, endometrial carcinoma, lung cancer, and osteosarcoma, expression of Fer1L4 was found to be decreased [12–16], whereas upregulation was reported in human glioblastoma and breast cancer [17, 18]. The detailed molecular and functional mechanism of its regulatory properties is yet unclear. In the absence of any data regarding to the role of Fer1L4 in renal-cell carcinoma (RCC) [17], our study was designed to explore the expression pattern of Fer1L4 in RCC and corresponding normal tissues to investigate its influence on tumor biology and impact on patients’ survival.

## Material and methods

### The cancer genome atlas (TCGA) cohort

Absolute RNA expression (RNAseq data) and clinical data from patients with clear-cell renal-cell carcinoma (ccRCC) were extracted from TCGA by TCGA browser (https://tcgabrowser.ethz.ch:3839/TEST/; v0.9.2). 603 samples of patients with available mRNA expression and clinical data were available (522 ccRCC and 81 normal).

### Validation cohort

We collected 103 renal tissue samples (69 ccRCC and 34 normal adjacent renal tissue) from patients who underwent radical or partial nephrectomy between 1997 and 2014 at the Department of Urology at the University Hospital Bonn. Normal adjacent renal tissue samples were taken from the normal tissue of the tumor-bearing removed kidney. Fresh-frozen tissues were stored at − 80 °C. Tissues were collected within the framework of the Biobank at the Center for Integrated Oncology Cologne-Bonn. The clinicopathological parameters of the patients are shown in Table 1.

**Table 1:** Clinicopathological parameters of study and TCGA cohort

	Study cohort		TCGA cohort	
	ccRCC *n* = 69 (%)	Normal *n* = 34 (%)	ccRCC *n* = 522 (%)	Normal *n* = 81 (%)
Sex				
Male	50 (72.5)	9 (26.5)	339 (64.9)	57 (70.3)
Female	19 (27.5)	25 (73.5)	183 (35.1)	24 (29.7)
Age				
Mean	65.7	64.4	60.62	63.06
Min–max	38–86	43–86	26–90	38–90
Pathological stage				
pT1	39	n.a	269	n.a
pT2	7	n.a	66	n.a
pT3	22	n.a	176	n.a
pT4	1	n.a	11	n.a
pN stage	14	n.a	15	n.a
pM-stage	2	n.a	81	n.a
Grading (WHO 2016)				
G1	10	n.a	14	n.a
G2	41	n.a	226	n.a
G3	14	n.a	206	n.a
G4	3	n.a	76	n.a

*n.a.* not applicable

### RNA isolation

Total RNA from fresh-frozen tissues was isolated using *mir*Vana™ miRNA Isolation Kit and then treated with DNA-*free*™ Kit (both Ambion, Foster City, CA, USA). After isolating the total RNA from cells using Total RNA Purification Mini Spin Kit (Genaxxon bioscience, Münster, Germany), the NanoDrop 2000 spectrophotometer was used to determine RNA quantity and quality (Thermo Fisher Scientific Wilmington, DE, USA). Additionally, RNA integrity of all samples was investigated by agarose gel electrophoresis. Afterwards, cDNA transcription was performed using 1 µg total RNA (PrimeScript™ RT reagent Kit with gDNA Eraser; Takara Bio, Saint-Germain-en-Laye, France).

### Real-time PCR

To determine the lncRNA expression profile of Fer1L4, we performed quantitative real-time PCR (qRT-PCR). Each qPCR was conducted with 5 ng/µl cDNA template and 10 pmol/µl of each forward and reverse primer using SYBR^®^ Premix Ex Taq™ II with ROX Plus (Takara Bio, Saint-Germain-en-Laye, France). PCR experiments were performed on an ABIPrism 7900 HT Fast Real-Time PCR System (Applied Biosystems, Foster City, CA, USA). The following primer sequences were used: Fer1L4 (forward ACA-CAG-TCC-TTG-TGG-GTT-CC; reverse CCT-GTC-TCC-TCC-ATC-TCT-CC). Obtained data were analyzed using Qbase + software (Biogazelle, Ghent, Belgium) with ACTB and PPIA as reference genes in the ${2}^{{ - \Delta \Delta C_{{\text{T}}} }}$ algorithm. Both genes were shown to be suitable reference genes for RCC studies [19, 20].

### Ethical approve

The study was approved by the local ethics committee (vote number: 045/17) and written pre-operative informed consent was obtained from all patients before enrollment.

### Statistical analyses

Statistical analyses (Mann–Whitney *U* test, Cox regression analyses) were performed using SPSS Statistics v24 (IBM, Ehningen, Germany). To calculate optimal cut-off values for lncRNA expression of study cohort, the survMisc package for R (based on consecutive evaluation of all available cut-offs using univariate Cox regression) was used.

## Results

### Analysis of the TCGA data

**Fig. 1:**
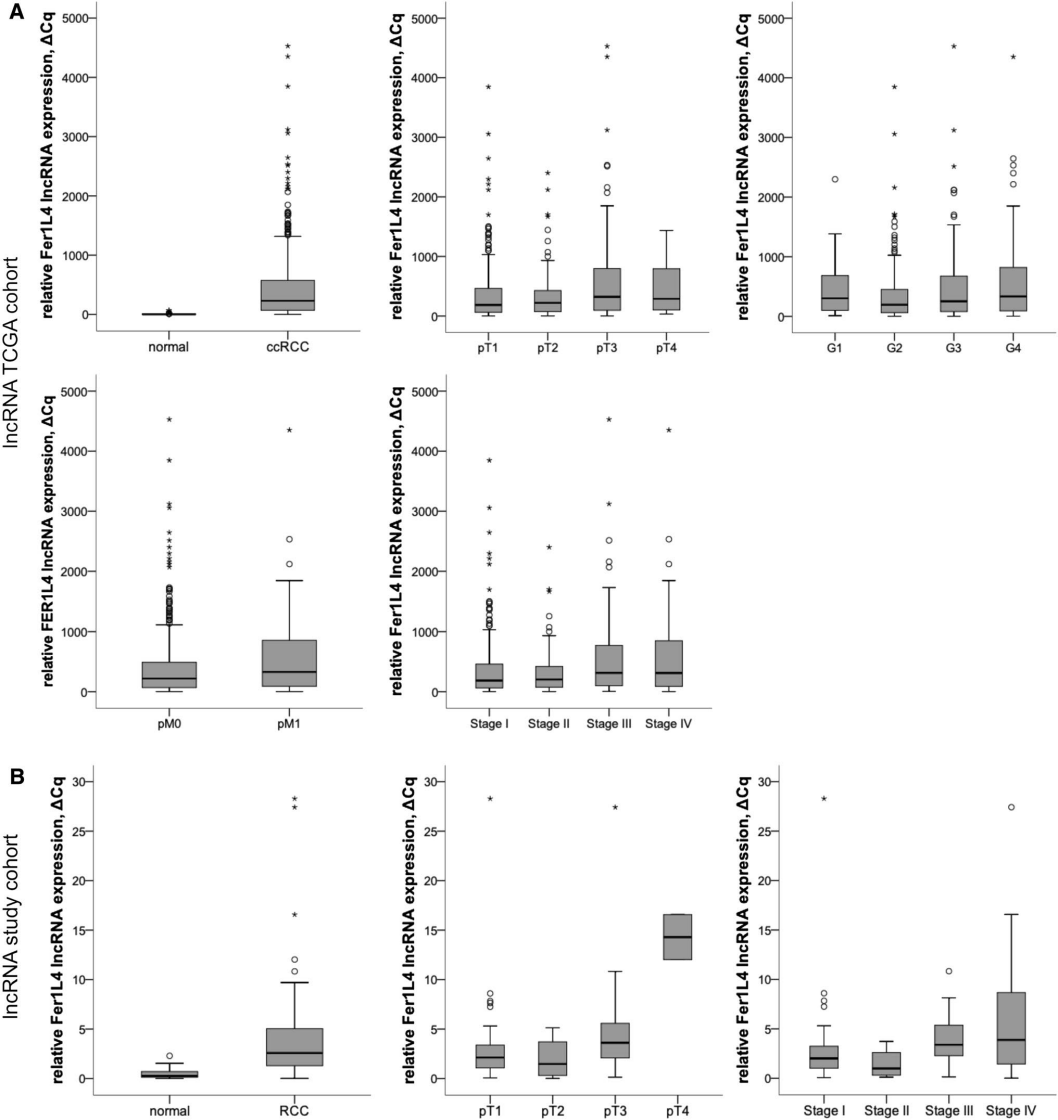
Fer1L4 lncRNA expression in normal renal tissue and clear-cell renal carcinoma (ccRCC). **a** Fer1L4 expression in the TCGA cohort: lncRNA expression is overexpressed in tissue of ccRCC compared to adjacent normal renal tissue (*p* < 0.001). Higher expression levels were observed in higher stage (*p* = 0.017), higher grade (*p* = 0.046), and metastatic ccRCC tumors (*p* = 0.034). **b** Fer1L4 expression in the validation cohort: confirmed overexpression in ccRCC tissue (*p* < 0.001). High expression levels were significantly associated with advanced disease stages (pT stage *p* = 0.009; AJCC stage *p* = 0.028)

**Fig. 2:**
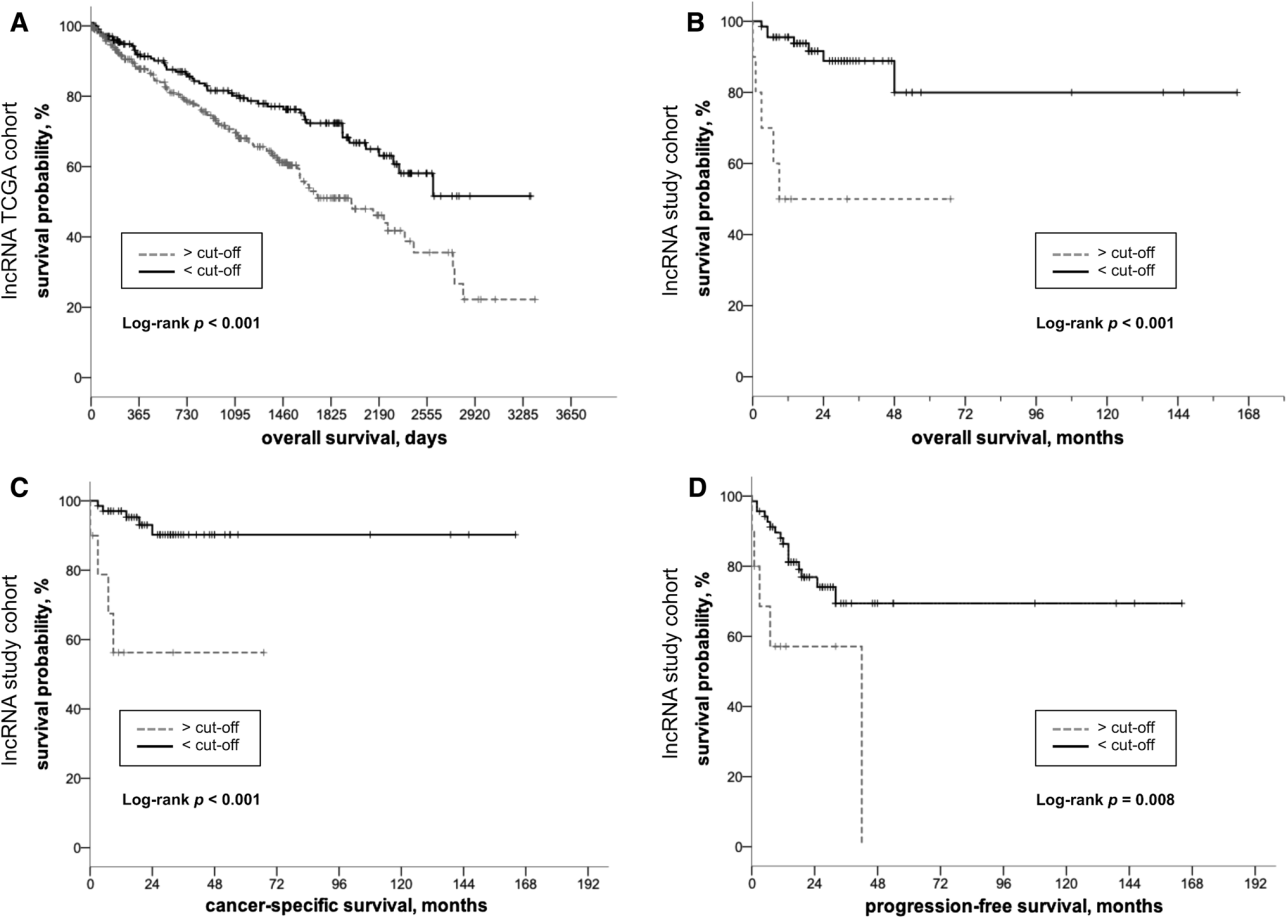
Kaplan–Meier estimates for Fer1L4 expression in patients with clear-cell renal-cell carcinoma. **a** TCGA cohort: Fer1L4 expression dichotomised using median as cut-off. Overexpression is predictive for overall survival (log rank *p* < 0.001). **b** Validation cohort: Fer1l4 expression dichotomised using optimized expression cut-off. Overexpression is predictive for overall survival (log rank *p* < 0.001), cancer-specific survival (log rank *p* < 0.001) and progression-free survival (log rank *p* = 0.008)

In the TCGA cohort, Fer1L4 expression levels were significantly higher in ccRCC tissues than in normal samples (*p* < 0.001, Fig. 1a). High lncRNA expression was positively correlated with indicators of tumor aggressiveness: pT stage (*p* = 0.017), AJCC stage (*p* = 0.014), grade (*p* = 0.046), and the presence of metastatic disease (*p* = 0.034, Fig. 1a). Increased Fer1L4 expression levels were negatively predictive for overall survival (OS) as determined using Kaplan–Meier estimates (log rank *p* < 0.001, Fig. 2a). Furthermore, univariate (HR 1.799, 95% CI 1.29–2.52, *p* = 0.001) and multivariate Cox regression analysis (HR 1.734, 95% CI 1.23–2.44, *p* = 0.002) indicated that increased Fer1L4 expression was a significant and independent predictor of shortened overall survival in patients with ccRCC. See Table 2.

**Table 2:** Cox regression analysis: overall survival in patients with ccRCC (lncRNA expression, TCGA cohort)

	Univariate analysis		Multivariate analysis	
	*p *value	HR (95% CI)	*p *value	HR (95% CI)
Fer1L4 expression^a^				
Low (< median)		1.0	–	1.0
High (> median)	0.001	1.799 (1.287–2.516)	0.002	1.734 (1.230–2.445)
pT stage				
pT1		1.0	–	1.0
pT2	0.158	1.497 (0.855–2.622)	0.957	0.984 (0.550–1.761)
pT3	< 0.001	3.619 (2.516–5.206)	0.010	1.767 (1.142–2.733)
pT4	< 0.001	12.307 (6.162–24.577)	0.202	1.877 (0.713–4.944)
pN stage				
pN0		1.0	–	1.0
pN1	0.001	3.229 (1.644–6.346)	0.523	1.315 (0.566–3.055)
pM stage				
pM0		1.0	–	1.0
pM1	< 0.001	4.592 (3.330–6.331)	< 0.001	2.309 (1.532–3.479)
Grading (WHO 2016)^b^				
G2		1.0	–	1.0
G3	0.002	1.886 (1.266–2.810)	0.030	1.576 (1.044–2.381)
G4	< 0.001	5.725 (3.756–8.726)	< 0.001	2.309 (1.532–3.479)

*HR* hazard ratio, *95% CI* 95% confidence interval

^a^Dichotomisation is based on median as cut-off for lncRNA expression

^b^G1 patients were excluded as they basically have an excellent survival and thus distorted the statistics for G2–4

### Validation of Fer1L4 expression as prognostic biomarker

To validate the findings from the TCGA cohort, ccRCC and normal adjacent renal tissues were examined by qPCR. As expected, we detected increased Fer1L4 levels in ccRCC compared to normal tissues (*p* < 0.001, Fig. 1b). Fer1L4 allowed discrimination of malignant and normal renal tissue with high sensitivity 73.2% and specificity 95.5% as determined using ROC analysis (area under curve 0.846, 95% CI 0.846–0.952). Additionally, Fer1L4 expression was associated with advanced disease: increased Fer1L4 expression was positively correlated with pT stage (*p* = 0.009, Fig. 1b) and AJCC-stage (*p* = 0.028, Fig. 1b). Finally, high Fer1L4 expression was predictive for progression-free survival (PFS, log rank *p* = 0.008), cancer-specific survival (CSS, log rank *p* < 0.001), and overall survival (OS, log rank *p* < 0.001); see Fig. 2b–d. Also, univariate (OS: HR 7.664, 95% CI 2.38–24.67, *p* = 0.001; CSS: HR 9.211, 95% CI 2.40–35.39, *p* = 0.001; PFS: HR 3.63, 95% CI 1.31–10.05, *p* = 0.013) and multivariate Cox regression analyses (OS: HR 7.06, 95% CI 1.88–26.44, *p* = 0.003; CSS: HR 10.686, 95% CI 2.21–51.61, *p* = 0.003, PFS: HR 3.56, 95% CI 1.19–10.62, *p* = 0.022) demonstrate an independent and significant value of Fer1L4 as prognostic biomarker in ccRCC patients (Tables 3, 4, 5), with higher expression meaning poor survival.

**Table 3:** Cox regression analysis: overall survival in patients with RCC (lncRNA expression, study cohort)

	Univariate analysis		Multivariate analysis	
	*p *value	HR (95% CI)	*p *value	HR (95% CI)
Fer1L4 expression^a^				
Low (< cut-off)		1.0	–	1.0
High (> cut-off)	0.001	7.664 (2.382–24.660)	0.003	7.067 (1.889–26.440)
pT stage^b^				
pT1		1.0	-	1.0
pT2	0.294	2.501 (0.451–13.861)	0.733	1.378 (0.216–8.765)
pT3 + pT4	0.482	1.577 (0.442–5.618)	0.989	0.989 (0.216–4.525)
pN stage^b^				
pN0		1.0	–	1.0
pN1	0.086	6.229 (0.772–50.250)	0.236	0.166 (0.008–3.241)
pM stage				
pM0		1.0	–	1.0
pM1	0.019	3.990 (1.256–12.671)	0.237	2.367 (0.565–9.902)
Grading (WHO 2016)^c^				
G2		1.0	–	1.0
G3	0.641	1.380 (0.356–5.354)	0.672	1.400 (0.294–6.664)
G4	0.016	7.806 (1.477–41.248)	0.032	16.420 (1.257–214.487)

*HR* hazard ratio, *95% CI* 95% confidence interval

^a^Dichotomisation is based on the best cut-off for lncRNA expression

^b^Despite the lack of significance based on the small data set, the parameter was included as an established prognostic parameter in the multivariate cox regression analyses

^c^G1 patients were excluded as they basically have an excellent survival and thus distorted the statistics for G2–4

**Table 4:** Cox regression analysis: cancer-specific survival in patients with RCC (lncRNA expression, study cohort)

	Univariate analysis		Multivariate analysis	
	*p *value	HR (95% CI)	*p *value	HR (95% CI)
Fer1L4 expression^a^				
Low (< cut-off)		1.0	–	1.0
High (> cut-off)	0.003	9.211 (2.398–35.383)	0.032	10.686 (2.212–51.614)
pT stage^b^				
pT1		1.0	–	1.0
pT2	0.607	1.813 (0.188–17.489)	0.908	0.870 (0.081–9.255)
pT3 + pT4	0.409	1.828 (0.436–7.667)	0.765	0.754 (0.118–4.810)
pN stage				
pN0		1.0	–	1.0
pN1	0.051	8.326 (0.992–69.898)	0.131	0.094 (0.004–2.025)
pM stage				
pM0		1.0	–	1.0
pM1	0.005	6.673 (1.805–25.335)	0.149	3.424 (0.643–18.234)
Grading (WHO 2016)^c^				
G2		1.0	–	1.0
G3	0.258	2.376 (0.53010.642)	0.327	2.458 (0.405–14.889)
G4	0.004	14.480 (2.348–89.284)	0.012	50.393 (2.360–1075.691)

*HR* hazard ratio, *95% CI* 95% confidence interval

^a^Dichotomisation is based on the best cut-off for lncRNA expression

^b^Despite the lack of significance based on the small data set, the parameter was included as an established prognostic parameter in the multivariate cox regression analyses

^c^G1 patients were excluded as they basically have an excellent survival and thus distorted the statistics for G2–4

**Table 5:** Cox regression analysis: progression-free survival in patients with RCC (lncRNA expression, study cohort)

	Univariate analysis		Multivariate analysis	
	*p *value	HR (95% CI)	*p *value	HR (95% CI)
Fer1L4 expression^a^				
Low (< cut-off)		1.0	–	1.0
High (> cut-off)	0.013	3.634 (1.314–10.053)	0.022	3.562 (1.194–10.622)
pT stage^b^				
pT1		1.0	–	1.0
pT2	0.499	1.740 (0.348–8.684)	0.868	1.150 (0.219–6.047)
pT3 + pT4	0.095	2.304 (0.864–6.141)	0.980	1.014 (0.321–3.202)
pN stage^b^				
pN0		1.0	–	1.0
pN1	0.256	3.241 (0.426–24.623)	0.185	0.142 (0.008–2.544)
pM-stage				
pM0		1.0	–	1.0
pM1	< 0.001	7.813 (3.283–18.596)	< 0.001	6.592 (2.172–20.007)
Grading (WHO 2016)^c^				
G2		1.0	–	1.0
G3	0.125	2.084 (0.816–5.322)	0.340	1.657 (0.586–4.684)
G4	0.038	5.193 (1.100–24.520)	0.124	6.170 (0.603–63.064)

*HR* hazard ratio, *95% CI* 95% confidence interval

^a^Dichotomisation is based on the best cut-off for lncRNA expression

^b^Despite the lack of significance based on the small data set, the parameter was included as an established prognostic parameter in the multivariate cox regression analyses

^c^G1 patients were excluded as they basically have an excellent survival and thus distorted the statistics for G2–4

## Discussion

More than 270,000 lncRNA transcripts exist in humans and their functional roles are poorly understood so far [5]. lncRNAs regulate gene expression at the level of chromatin modification, transcription, and post-transcriptional processing [21–23]. Furthermore, several studies have described a potential oncogenic role in various malignancies including renal-cell carcinoma [9, 10].

To get a better understanding of ccRCC pathogenesis, we studied Fer1L4 expression in the TCGA dataset as well as in an independent ccRCC cohort from the University Hospital Bonn. Fer1L4 expression was increased in study cohort in ccRCC tissue compared to normal renal tissue. In addition, Fer1L4 expression has the potential as prognostic tissue biomarker for ccRCC: increased levels were observed in advanced staged and less-differentiated ccRCCs. Furthermore, Fer1L4 expression was an independent predictor of PFS, CSS, and OS in ccRCC patients. A recent study analyzed Fer1L4 in a pan-cancer study and demonstrated similar findings for a subset of RCC patients [17].

Earlier studies investigated the expression and possible influence of Fer1L4 on tumorigenesis in various other tumor entities. Despite the general assumption of its function as a tumor suppressor, both underexpression and overexpression of Fer1L4 have been described. In colon and gastric carcinoma, Fer1L4 exerted a tumor suppressive effect as ceRNA which regulates the expression of PTEN via the release of miR-106-5p [11, 24–26]. Studies in osteosarcoma, lung cancer, and hepatocellular cancer indicated that Fer1L4 suppresses proliferation and induces apoptosis in vivo and in vitro by inhibiting the Pi3K/AKT pathway [16, 27–29]. However, compared to this, other experiments demonstrated oncogenic properties of Fer1L4. In glioma cells, the regulation of miR-372 and E2F1 appeared to be one molecular mechanism of the oncogenic effect. Fer1L4 acted as a ceRNA that interacts with miR-372 and thus upregulates E2F1 resulting in promotion of cell cycle and proliferation [30]. Additionally, siRNA-mediated knockdown of Fer1L4 decreased invasiveness of glioblastoma cells and induced apoptosis [18]. In breast cancer, upregulation of Fer1L4 is linked to promoter hypomethylation suggesting that Fer1L4 expression is epigenetically regulated [17]. Taken together, Fer1L4 expression is different in various tumor entities and may exercise as well as oncogenic and tumor suppressive effects. This heterogenic influence of Fer1L4 on oncogenesis shows that its function on tumor biology is yet incompletely understood.

Our work presents a comprehensive expression analysis of Fer1L4 in clear-cell renal-cell carcinoma and its influence on clinicopathological parameters and survival highlighting the role of Fer1L4 as a prognostic tissue biomarker. Though, for a precise evaluation of the influence on tumor biology of ccRCC, functional analyses under consideration of the underlying signal pathways are mandatory.

## Conclusion

Our study shows that the lncRNA Fer1L4 is significantly overexpressed in ccRCC tissue, and that high Fer1L4 expression levels are indicative for tumor aggressiveness and cancer recurrence. Furthermore, Fer1L4 is a prognostic biomarker for patients with ccRCC that appears to exert properties as an oncogene. Further functional investigations are warranted to investigate Fer1L4 molecular mechanisms in ccRCC and its value as a potential therapeutic target.

## Abbreviations

ccRCC: Clear-cell renal-cell carcinoma
ceRNA: Competing endogenous RNA
Fer1L4: Fer-1 like family member 4
lncRNA: Long non-coding RNA
qPCR: Quantitative polymerase chain reaction
ROC: Receiver operator characteristics
